# Supplementary figures and images for: Cell-intrinsic mTOR/LET-363 influences morphological aging of the ALM touch receptor neuron in Caenorhabditis elegans
Source: PLoS One. 2026 Mar 27;21(3):e0345575. doi: 10.1371/journal.pone.0345575 (PMC13028374; doi:10.1371/journal.pone.0345575)

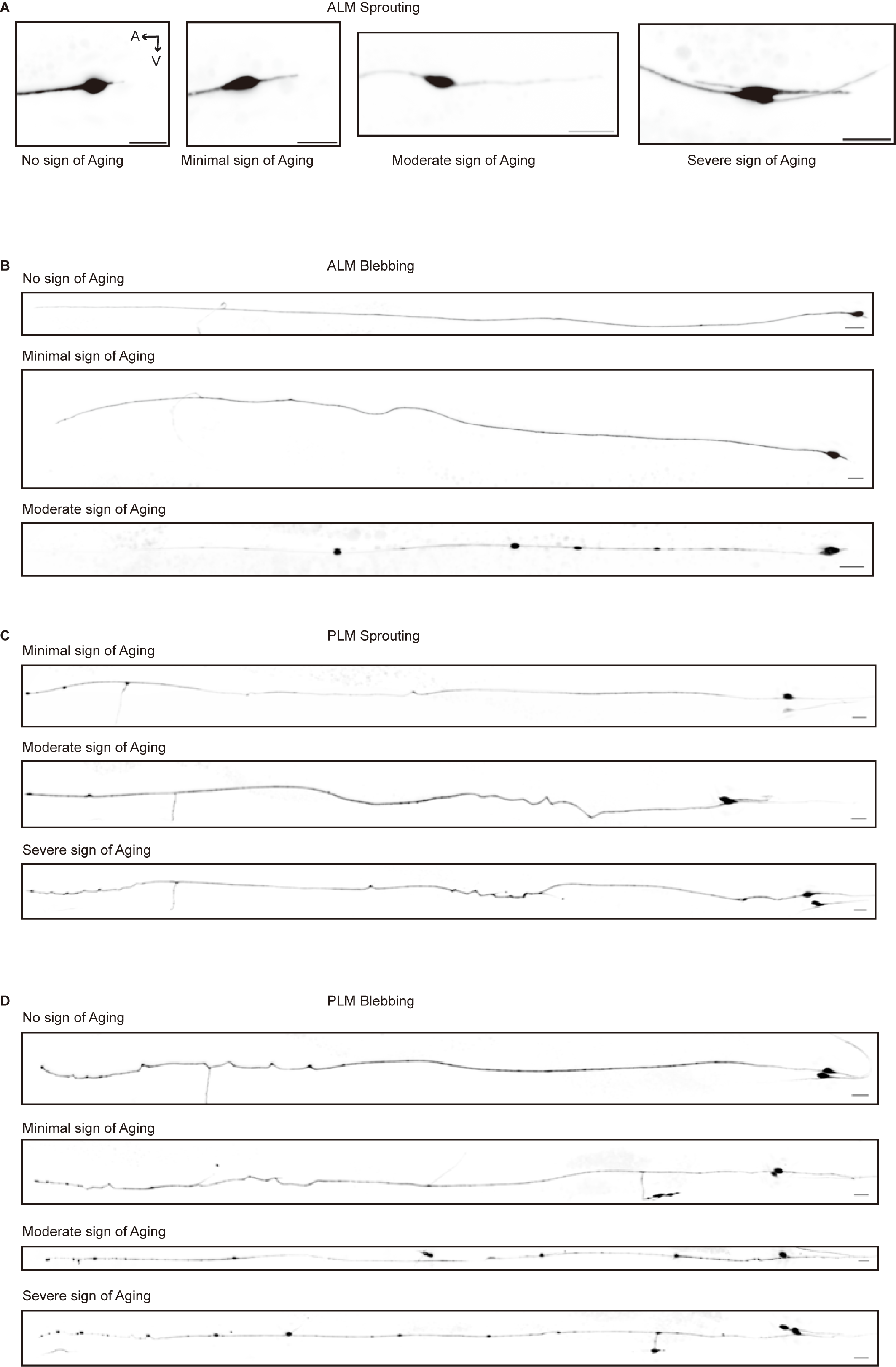

Supplement: S1 Fig — TRNs were visualized with zdIs5[PTRN > GFP] at Day 12 of adulthood in strain PBT206 zdIs5 let-363(wy1706); tmIs1075. Scale: 10 µm. (TIF) [file pone.0345575.s001.tif]

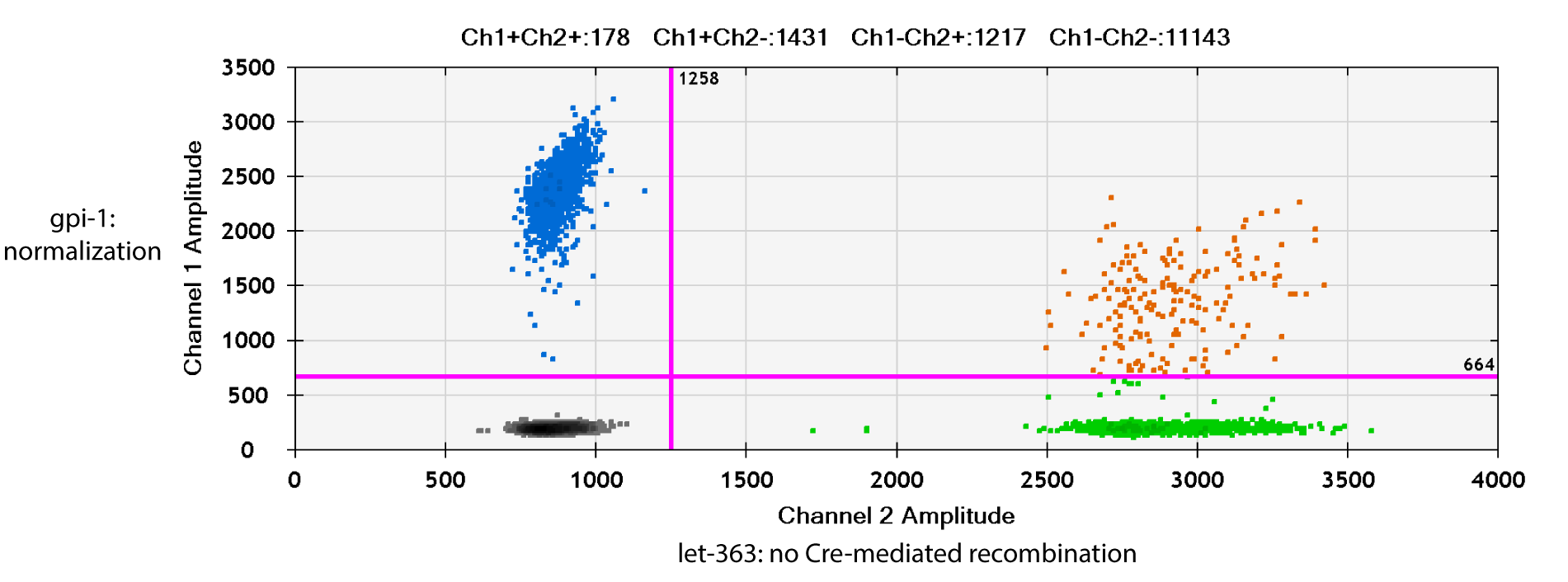

Supplement: S2 Fig — (TIF) [file pone.0345575.s002.tif]
