# Supplementary material for: Cell-intrinsic mTOR/LET-363 influences morphological aging of the ALM touch receptor neuron in Caenorhabditis elegans
Source: PLoS One. 2026 Mar 27;21(3):e0345575. doi: 10.1371/journal.pone.0345575 (PMC13028374; doi:10.1371/journal.pone.0345575)
Supplement: Attachment — Submitted filename: Review PONE-D-25-63170 _2025.docx [file pone.0345575.s007.docx]

Review:

|  | PONE-D-25-63170 |
| --- | --- |

The work focuses on the question of whether mTor functions in a neuron-specific manner (as opposed to other cell types) to control aging / They a generated post-developmental knockdown of mTor (let-363) (which is in the mTor1 and mTor2 complex) in the touch neurons. The allele is lethal, so constructing a post-development knockout, that is specific to touch neurons, is important for the study and is a strength of the study. This study is also important to address the discrepancy of mTor knockdown and observed phenotypes: somatic knockdown results in a shortened lifespan, but pan neuronal knockdown extends the lifespan.

Introduction:

Well written and sets up the study nicely. One fundamental question, perhaps outside of the study but important to acknowledge and consider, is that the mTorc1 and mTorc2 complexes in C. elegans and mammals have been shown to regulate specific and sometime separate outcomes neurons. A let-363 knockdown will alter both complexes and will perhaps make the results harder to interpret.

Methods:

I am curious to know why the let-363(1706) worms were only included in 2 replicates while the rest were included in 3.

This sentence could use clarity: “Every 2 days, worms were transferred to new NGM plates with E. coli OP50 to prevent larvae from growing to full adults.” What I think you mean is that the adult worms were transferred so they would not be contaminated by the growing prodigy and thus you would be able to track the original population.

Very nice chart describing how you are quantifying your phenotypes in the worms.

I think it would be important to clarify throughout that “post-developmental knockout” in this case means throughout. I had trouble following the groups and when they were heat shocked.

I note you scored the neurons at day 12. Did you look at them earlier to make sure neuronal development was not impaired I the groups heat shocked as eggs?

Results and discussion:

The authors split the hypothesis into 2 sets of experiments:

1. Let-363 knockdown pan-somatically in adulthood
2. Neuron specific let-363 knockdown.

You mention that the neurons have invariant morphology in young, wild-type adults. Does this changes with either of the knockdowns that you have described?

Figure 1

This looks at pan-somatic knockdown of let-363 (using let-363(wy1706) and it does not robustly alter lifespan or neuron morphological again. This figure legend could be read as the animals were heat shocked at day 1 of adulthood, but the methods suggest that you heat shocked eggs. I do see that you clarified in (B), but I would recommend clarifying the ages of the worms and when they are heat shocked throughout.

I see that neuron morphological aging is quantified at day 12 in the figure legend. It might be useful to indicate that in the figure for easy.

When you knockdown let-363 post-developmentally, when is the heat shock occuring? I would keep this clear throughout for ease. I can see day 0 buried in the paragraph, but it would be useful to keep it clear.

“This lifespan reduction is not robust in our paradigm, however, as it occurred in only one of two biological replicates” It might be useful to run another replicate or two to tease this out. Is there a reason you did not?

“We cannot exclude the possibility that the heat-shock, or post developmental pan-somatic let-363 knockdown, provides a benefit for neuron morphological aging that manifests earlier or later in life.” It could also be off target effects because you saw this in several of the heat shock groups and you mentioned that other labs see the same thing.

Neuron specific knockdown. No robust change in lifespan, as expected. Neuron intrinsic knockdown of let-363 resulted in significant reduction in ALM blebbing. It seems like you did not get robust knockdown using this method. Perhaps that could be a reason there are not more changes in phenotypes.

Interpretation of results is reasonable. References are good.

Overall a very good manuscript, I recommend minor edits for clarity.
